# From approximate to exact integer programming

**DOI:** 10.1007/s10107-024-02084-1

**Published:** 2024-04-24

**Authors:** Daniel Dadush, Friedrich Eisenbrand, Thomas Rothvoss

**Affiliations:** 1 Centrum Wiskunde & Informatica (CWI), Amsterdam, The Netherlands; 2 École Polytechnique Fédérale de Lausanne (EPFL), Lausanne, Switzerland; 3 University of Washington, Seattle, WA USA

**Keywords:** Integer programming, Lattices, Convex geometry, 15A, 52B, 52C, 68Q, 68R, 68W, 90B, 90C

## Abstract

Approximate integer programming is the following: For a given convex body $K \subseteq {\mathbb {R}}^n$, either determine whether $K \cap {\mathbb {Z}}^n$ is empty, or find an integer point in the convex body $2\cdot (K - c) +c$ which is *K*, scaled by 2 from its center of gravity *c*. Approximate integer programming can be solved in time $2^{O(n)}$ while the fastest known methods for exact integer programming run in time $2^{O(n)} \cdot n^n$. So far, there are no efficient methods for integer programming known that are based on approximate integer programming. Our main contribution are two such methods, each yielding novel complexity results. First, we show that an integer point $x^* \in (K \cap {\mathbb {Z}}^n)$ can be found in time $2^{O(n)}$, provided that the *remainders* of each component $x_i^* \mod \ell$ for some arbitrarily fixed $\ell \ge 5(n+1)$ of $x^*$ are given. The algorithm is based on a *cutting-plane technique*, iteratively halving the volume of the feasible set. The cutting planes are determined via approximate integer programming. Enumeration of the possible remainders gives a $2^{O(n)}n^n$ algorithm for general integer programming. This matches the current best bound of an algorithm by Dadush (Integer programming, lattice algorithms, and deterministic, vol. Estimation. Georgia Institute of Technology, Atlanta, 2012) that is considerably more involved. Our algorithm also relies on a new *asymmetric approximate Carathéodory theorem* that might be of interest on its own. Our second method concerns integer programming problems in equation-standard form $Ax = b, 0 \le x \le u, \, x \in {\mathbb {Z}}^n$. Such a problem can be reduced to the solution of $\prod _i O(\log u_i +1)$ approximate integer programming problems. This implies, for example that *knapsack* or *subset-sum* problems with *polynomial variable range* $0 \le x_i \le p(n)$ can be solved in time $(\log n)^{O(n)}$. For these problems, the best running time so far was $n^n \cdot 2^{O(n)}$.

## Introduction

Many *combinatorial optimization problems* as well as many problems from the *algorithmic geometry of numbers* can be formulated as an *integer linear program*

1 $$\begin{aligned} \max \{ \left<c,x\right> \mid Ax\le b, x \in {\mathbb {Z}}^n\} \end{aligned}$$

where $A \in {\mathbb {Z}}^{m \times n}, b\in {\mathbb {Z}}^m$ and $c \in {\mathbb {Z}}^n$, see, e.g. [1–3]. Lenstra [4] has shown that integer programming can be solved in polynomial time, if the number of variables is fixed. A careful analysis of his algorithm yields a running time of $2^{O(n^2)}$ times a polynomial in the binary encoding length of the input of the integer program. Kannan [5] has improved this to $n^{O(n)}$, where, from now on we ignore the extra factor that depends polynomially on the input length. At the time this paper was first submitted, the best algorithm was the one of Dadush [6] with a running time of $2^{O(n)} \cdot n^n$.

The question whether there exists a *singly exponential time*, i.e., a $2^{O(n)}$-algorithm for integer programming is one of the most prominent open problems in the area of algorithms and complexity. Integer programming can be described in the following more general form. Here, a *convex body* is synonymous for a full-dimensional compact and convex set.
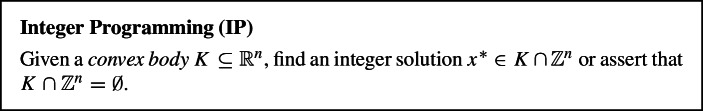


The convex body *K* must be well described in the sense that there is access to a *separation oracle*, see [1]. Furthermore, one assumes that *K* contains a ball of radius $r>0$ and that it is contained in some ball of radius *R*. In this setting, the current best running times hold as well. The additional polynomial factor in the input encoding length becomes a polynomial factor in $\log (R/r)$ and the dimension *n*. Central to this paper is *Approximate integer programming* which is as follows.
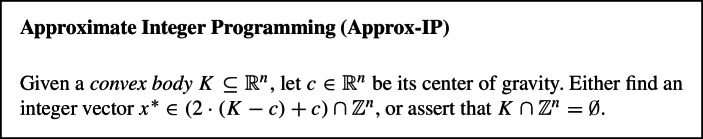


The convex body $2 \cdot (K-c) + c$ is *K* scaled by a factor of 2 from its center of gravity. The algorithm of Dadush [7] solves approximate integer programming in singly exponential time $2^{O(n)}$. Despite its clear relation to exact integer programming, there is no reduction from exact to approximate known so far. Our guiding question is the following: Can approximate integer programming be used to solve the exact version of (specific) integer programming programming problems?

### Contributions of this paper

We present two different algorithms to reduce the exact integer programming problem (IP) to the approximate version (Approx-IP).

- Our first method is a randomized cutting-plane algorithm that, in time $2^{O(n)}$ and for any $\ell \ge 5(n+1)$ finds a point in $K\cap ( {\mathbb {Z}}^n/ \ell )$ with high probability, if *K* contains an integer point. This algorithm uses an oracle for (Approx-IP) on *K* intersected with one side of a hyperplane that is close to the center of gravity. Thereby, the algorithm collects $\ell$ integer points close to *K*. The collection is such that the convex combination with uniform weights $1/\ell$ of these points lies in *K*. If, during an iteration, no point is found, the volume of *K* is roughly halved and eventually *K* lies on a lower-dimensional subspace on which one can recurse.
- If equipped with the component-wise remainders $v \equiv x^* \pmod {\ell }$ of a solution $x^*$ of (IP), one can use the algorithm to find a point in $(K - v) \cap {\mathbb {Z}}^n$ and combine it with the remainders to a full solution of (IP), using that $(K-v) \cap \ell \mathbb {Z}^n \ne \emptyset$. This runs in singly exponential randomized time $2^{O(n)}$. Via enumeration of all remainders, one obtains an algorithm for (IP) that runs in time $2^{O(n)} \cdot n^n$. This matches the best-known running time for general integer programming [7], which is considerably involved.
- Our analysis depends on a new *approximate Carathéodory theorem* that we develop in Sect. 4. While approximate Carathéodory theorems are known for centrally symmetric convex bodies [8–10], our version is for general convex sets and might be of interest on its own.
- Our second method is for integer programming problems $Ax = b,\, x \in {\mathbb {Z}}^n, \, 0 \le x \le u$ in equation standard form. We show that such a problem can be reduced to $2^{O(n)} \cdot (\prod _i \log (u_i+1))$ instances of (Approx-IP). This yields a running time of $(\log n)^{O(n)}$ for such IPs, in which the variables are bounded by a polynomial in the dimension. The so-far best running time for such instances was $2^{O(n)} \cdot n^n$ at the time of the first submission of this paper. Well known benchmark problems in this setting are *knapsack* and *subset-sum* with polynomial upper bounds on the variables, see Sect. 5.

### Related work

If the convex body *K* is an ellipsoid, then the integer programming problem (IP) is the well known *closest vector problem (CVP)* which can be solved in time $2^{O(n)}$ with an algorithm by Micciancio and Voulgaris [11]. Blömer and Naewe [12] previously observed that the sampling technique of Ajtai et al. [13] can be modified in such a way as to solve the closest vector approximately. More precisely, they showed that a $(1+\epsilon )$-approximation of the closest vector problem can be found in time $O(2 + 1/\epsilon )^n$ time. This was later generalized to arbitrary convex sets by Dadush [7]. This algorithm either asserts that the convex body *K* does not contain any integer points, or it finds an integer point in the body stemming from *K* scaled by $(1+\epsilon )$ from its center of gravity. Also the running time of this randomized algorithm is $O(2 + 1/\epsilon )^n$. In our paper, we restrict to the case $\epsilon = 1$ which can be solved in singly exponential time. The technique of reflection sets was also used by Eisenbrand et al. [14] to solve (CVP) in the $\ell _\infty$-norm approximately in time $O(2 + \log (1/\epsilon ))^n$.

In the setting in which integer programming can be attacked with dynamic programming, tight upper and lower bounds on the complexity are known [15–17]. Our $n^n \cdot 2^{O(n)}$ algorithm could be made more efficient by constraining the possible remainders of a solution $\pmod {\ell }$ efficiently. This barrier is different than the one in classical integer-programming methods that are based on branching on flat directions [1, 4] as they result in a branching tree of size $n^{O(n)}$.

The *subset-sum problem* is as follows. Given a set $Z \subseteq {\mathbb {N}}$ of *n* positive integers and a *target value* $t \in {\mathbb {N}}$, determine whether there exists a subset $S \subseteq Z$ with $\sum _{ s \in S} s = t$. Subset sum is a classical NP-complete problem that serves as a benchmark in algorithm design. The problem can be solved in pseudopolynomial time [18] by dynamic programming. The current fastest pseudopolynomial-time algorithm is the one of Bringmann [19] that runs in time $O( n+t)$ up to polylogarithmic factors. There exist instances of subset-sum whose set of feasible solutions, interpreted as 0/1 incidence vectors, require numbers of value $n^n$ in the input, see [20]. Lagarias and Odlyzko [21] have shown that instances of subset sum in which each number of the input *Z* is drawn uniformly at random from $\{1,\dots ,2^{O(n^2)}\}$ can be solved in polynomial time with high probability. The algorithm of Lagarias and Odlyzko is based on the LLL-algorithm [22] for lattice basis reduction.

### Subsequent work

After the acceptance of the conference version of this work, Reis and Rothvoss [23] proved that an algorithm originally suggested by Dadush [6] can solve any *n*-variable integer program $\max \{ \left<c,x \right> \mid Ax \le b, x \in \mathbb {Z}^n \}$ in time $(\log n)^{O(n)}$ times a polynomial in the encoding length of *A*, *b* and *c*. However, the question whether there is a $2^{O(n)}$-time algorithm remains wide open and the approach used by Reis and Rothvoss inherently cannot provide running times below $(\log n)^{O(n)}$.

## Preliminaries

A *lattice* $\Lambda$ is the set of integer combinations of linearly independent vectors, i.e. $\Lambda := \Lambda (B):= \{ Bx \mid x \in \mathbb {Z}^r \}$ where $B \in \mathbb {R}^{n \times r}$ has linearly independent columns. The *determinant* is the volume of the *r*-dimensional parallelepiped spanned by the columns of the basis *B*, i.e. $\det (\Lambda ):= \sqrt{\det _r(B^TB)}$. We say that $\Lambda$ has *full rank* if $n=r$. In that case the determinant is simply $\det (\Lambda )=|\det _n(B)|$. For a full rank lattice $\Lambda$, we denote the dual lattice by $\Lambda ^* = \{ y \in \mathbb {R}^n \mid \left<x,y\right> \in \mathbb {Z}\;\forall x \in \Lambda \}$. Note that $\det (\Lambda ^*) \cdot \det (\Lambda ) = 1$. For an introduction to lattices, we refer to [24].

A set $Q \subseteq \mathbb {R}^n$ is called a *convex body* if it is convex, compact and has a non-empty interior. A set *Q* is *symmetric* if $Q = -Q$. Recall that any symmetric convex body *Q* naturally induces a norm $\Vert \cdot \Vert _Q$ of the form $\Vert x\Vert _Q = \min \{ s \ge 0 \mid x \in sQ\}$. For a full rank lattice $\Lambda \subseteq \mathbb {R}^n$ and a symmetric convex body $Q \subseteq \mathbb {R}^n$ we denote $\lambda _1(\Lambda ,Q):= \min \{ \Vert x\Vert _Q \mid x \in \Lambda {\setminus } \{ \varvec{0}\} \}$ as the length of the shortest vector with respect to the norm induced by *Q*. We denote the Euclidean ball by $B_2^n:= \{ x \in \mathbb {R}^n \mid \Vert x\Vert _2 \le 1\}$ and the $\ell _\infty$-ball by $B_{\infty }^n:= [-1,1]^n$. An (origin centered) *ellipsoid* is of the form  where $A: \mathbb {R}^n \rightarrow \mathbb {R}^n$ is an invertible linear map. For any such ellipsoid  there is a unique positive definite matrix $M \in \mathbb {R}^{n \times n}$ so that . The *barycenter* (or *centroid*) of a convex body *Q* is the point $\frac{1}{\text {Vol}_n(Q)}\int _Q x \; dx$. We will use the following version of (Approx-IP) that runs in time $2^{O(n)}$, provided that the symmetrizer for the used center *c* is large enough. This is the case for *c* being the center of gravity, see Theorem 5. Note that the center of gravity of a convex body can be (approximately) computed in randomized polynomial time [25, 26].

### Theorem 1

(Dadush [7]) There is a $2^{O(n)}$-time algorithm $\textsc {ApxIP}(K,c,\Lambda )$ that takes as input a convex body $K \subseteq \mathbb {R}^n$, a point $c \in K$ and a lattice $\Lambda \subseteq \mathbb {R}^n$. Assuming that $\text {Vol}_n( (K-c) \cap (c-K) ) \ge 2^{-\Theta (n)} \text {Vol}_n(K)$ the algorithm either returns a point $x \in (c+2(K-c)) \cap \Lambda$ or returns empty if $K \cap \Lambda = \emptyset$.

One of the classical results in the geometry of numbers is Minkowski’s Theorem which we will use in the following form:

### Theorem 2

(Minkowski’s Theorem) For a full rank lattice $\Lambda \subseteq \mathbb {R}^n$ and a symmetric convex body $Q \subseteq \mathbb {R}^n$ one has

$$\begin{aligned} \lambda _1(\Lambda ,Q) \le 2 \cdot \Big (\frac{\det (\Lambda )}{\text {Vol}_n(Q)}\Big )^{1/n} \end{aligned}$$

We will use the following bound on the density of sublattices which is an immediate consequence of Minkowski’s Second Theorem. Here we abbreviate $\lambda _1(\Lambda ):= \lambda _1(\Lambda ,B_2^n)$.

### Lemma 3

Let $\Lambda \subseteq \mathbb {R}^n$ be a full rank lattice. Then for any *k*-dimensional sublattice ${\tilde{\Lambda }} \subseteq \Lambda$ one has $\det ({\tilde{\Lambda }}) \ge (\frac{\lambda _1(\Lambda )}{\sqrt{k}})^k$.

Finally, we revisit a few facts from *convex geometry*. Details and proofs can be found in the excellent textbook by Artstein-Avidan, Giannopoulos and Milman [27].

### Lemma 4

(Grünbaum’s Lemma) Let $K \subseteq \mathbb {R}^n$ be any convex body and let $\left<a,x\right> = \beta$ be any hyperplane through the barycenter of *K*. Then $\frac{1}{e} \text {Vol}_n(K) \le \text {Vol}_n(\{ x \in K \mid \left<a,x\right> \le \beta \}) \le (1-\frac{1}{e}) \text {Vol}_n(K)$.

For a convex body *K*, there are two natural symmetric convex bodies that approximate *K* in many ways: the “inner symmetrizer” $K \cap (-K)$ (provided $\varvec{0} \in K$) and the “outer symmetrizer” in form of the *difference body* $K-K$. The following is a consequence of a more general inequality of Milman and Pajor.

### Theorem 5

Let $K \subseteq \mathbb {R}^n$ be any convex body with barycenter $\varvec{0}$. Then $\text {Vol}_n(K \cap (-K)) \ge 2^{-n}\text {Vol}_n(K)$.

In particular Theorem 5 implies that choosing *c* as the barycenter of *K* in Theorem 1 results in a $2^{O(n)}$ running time — however this will not be the choice that we will later make for *c*. Also the size of the difference body can be bounded:

### Theorem 6

(Inequality of Rogers and Shephard) For any convex body $K \subseteq \mathbb {R}^n$ one has $\text {Vol}_n(K-K) \le 4^n \text {Vol}_n(K)$.

Recall that for a convex body *Q* with $\varvec{0} \in \text {int}(Q)$, the *polar* is $Q^{\circ } = \{ y \in \mathbb {R}^n \mid \left<x,y\right> \le 1 \; \forall x \in Q\}$. We will use the following relation between volume of a symmetric convex body and the volume of the polar; to be precise we will use the lower bound (which is due to Bourgain and Milman).

### Theorem 7

(Blaschke-Santaló-Bourgain-Milman) For any symmetric convex body $Q \subseteq \mathbb {R}^n$ one has

$$\begin{aligned} C^n \le \frac{\text {Vol}_n(Q) \cdot \text {Vol}_n(Q^{\circ })}{\text {Vol}_n(B_2^n)^2} \le 1 \end{aligned}$$

where $C>0$ is a universal constant.

We will also rely on the result of Frank and Tardos to reduce the bit complexity of constraints:

### Theorem 8

(Frank, Tardos [28]) There is a polynomial time algorithm that takes $(a,b) \in \mathbb {Q}^{n+1}$ and $\Delta \in \mathbb {N}_+$ as input and produces a pair $({\tilde{a}},{\tilde{b}}) \in \mathbb {Z}^{n+1}$ with $\Vert {\tilde{a}}\Vert _{\infty },|{\tilde{b}}| \le 2^{O(n^3)} \cdot \Delta ^{O(n^2)}$ so that $\left<a,x\right> = b \Leftrightarrow \left<{\tilde{a}},x\right> = {\tilde{b}}$ and $\left<a,x\right> \le b \Leftrightarrow \left<{\tilde{a}},x\right> \le {\tilde{b}}$ for all $x \in \{ -\Delta ,\ldots ,\Delta \}^n$.

## The cut-or-average algorithm

**Fig. 1:**
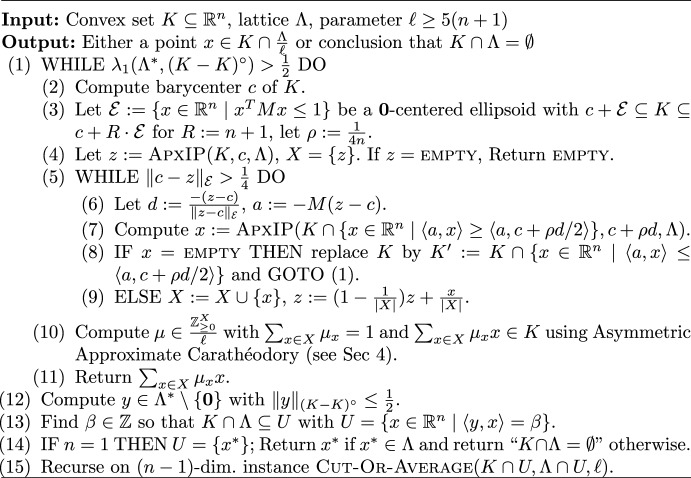
The Cut-Or-Average algorithm

First, we discuss our $\textsc {Cut-Or-Average}$ algorithm that on input of a convex set *K*, a lattice $\Lambda$ and integer $\ell \ge 5(n+1)$, either finds a point $x \in \frac{\Lambda }{\ell } \cap K$ or decides that $K \cap \Lambda = \emptyset$ in time $2^{O(n)}$. Note that for any polyhedron $K = \{ x \in \mathbb {R}^n \mid Ax \le b\}$ with rational *A*, *b* and lattice $\Lambda$ with basis *B* one can compute a value of $\Delta$ so that $\log (\Delta )$ is polynomial in the encoding length of *A*, *b* and *B* and $K \cap \Lambda \ne \emptyset$ if and only if $K \cap [-\Delta ,\Delta ]^n \cap \Lambda \ne \emptyset$. See Schrijver [29] for details. In other words, w.l.o.g. we may assume that our convex set is bounded. The pseudo code of the algorithm can be found in Fig. 1.

An intuitive description of the algorithm is as follows: we compute the barycenter *c* of *K* and an ellipsoid  that approximates *K* up to a factor of $R = n+1$.

**Fig. 2:**
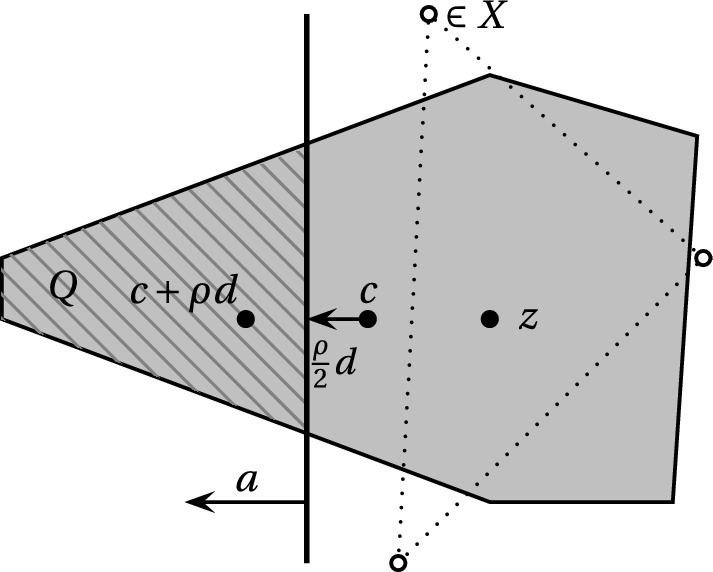
Visualization of the inner WHILE loop where $Q:= K \cap \{ x \in \mathbb {R}^n \mid \left<a,x\right> \ge \left<a,c+\frac{\rho }{2}d\right>\}$

The goal is to find a point *z* close to the barycenter *c* so that *z* is a convex combination of lattice points that all lie in a 3-scaling of *K*. We begin by choosing *z* as any such lattice vector and then iteratively update *z* using the oracle for approximate integer programming from Theorem 1 to move closer to *c* (Fig. 2).

If this succeeds, then we can directly use an asymmetric version of the *Approximate Carathéodory Theorem* (Lemma 18) to find an unweighted average of $\ell$ lattice points that lies in *K*; this would be a vector of the form $x \in \frac{\Lambda }{\ell } \cap K$. If the algorithm fails to approximately express *c* as a convex combination of lattice points, then we will have found a hyperplane *H* going almost through the barycenter *c* so that $K \cap H_{\ge }$ does not contain a lattice point. Then the algorithm continues searching in $K \cap H_{\le }$. This case might happen repeatedly, but after polynomial number of times, the volume of *K* will have dropped below a threshold so that we may recurse on a *single* $(n-1)$-dimensional subproblem. We will now give the detailed analysis. Note that in order to obtain a clean exposition we did not aim to optimize any constant. However by merely tweaking the parameters one could make the choice of $\ell = (1+\varepsilon )n$ work for any constant $\varepsilon > 0$.

### Bounding the number of iterations

We begin the analysis with a few estimates that will help us to bound the number of iterations.

#### Lemma 9

Any point *x* found in line (7) lies in a 3-scaling of *K* around *c*, i.e. $x \in c + 3(K-c)$ assuming $0 <\rho \le 1$.

#### Proof

We verify that

$$\begin{aligned} x \in (c-\rho d) + 2(K-(c-\rho d)) = c + 2(K-c) + \rho d \subseteq c + 3(K-c) \end{aligned}$$

using that . $$\square $$

Next we bound the distance of *z* to the barycenter:

#### Lemma 10

At the beginning of the *k*th iterations of the WHILE loop on line (5), one has .

#### Proof

We prove the statement by induction on *k*. At $k=1$, by construction on line (4), . Thus , as needed.

Now assume $k \ge 2$. Let $z,z'$ denote the values of *z* during iteration $k-1$ before and after the execution of line (9) respectively, and let *x* be the vector found on line (7) during iteration $k-1$. Note that $z' = (1-\frac{1}{k}) z + \frac{1}{k} x$. By the induction hypothesis, we have that . Our goal is to show that . In (6), we define *d* as the normalized version of $z-c$ with  and hence $d \in K-c$. By construction $\langle a, x-c \rangle \ge 0$ and from Lemma 9 we have $x \in c + 3(K-c)$ which implies . The desired bound on the -norm of $z'-c$ follows from the following calculation:$\square$

In particular Lemma 10 implies an upper bound on the number of iterations of the inner WHILE loop:

#### Corollary 11

The WHILE loop on line (5) never takes more than $36R^2$ iterations.

#### Proof

By Lemma 10, for $k:= 36R^2$ one has . $\square$

Next, we prove that every time we replace *K* by $K' \subset K$ in line (8), its volume drops by a constant factor.

#### Lemma 12

In step (8) one has $\text {Vol}_n(K') \le (1-\frac{1}{e}) \cdot (1+\frac{\rho }{2})^n \cdot \text {Vol}_n(K)$ for any $\rho \ge 0$. In particular for $0 \le \rho \le \frac{1}{4n}$ one has $\text {Vol}_n(K') \le \frac{3}{4} \text {Vol}_n(K)$.

#### Proof

The claim is invariant under affine linear transformations, hence we may assume w.l.o.g. that , $M = I_n$ and $c=\varvec{0}$. Note that then $B_2^n \subseteq K \subseteq R B_2^n$. Let us abbreviate $K_{\le t}:= \{ x \in K \mid \left<d,x\right> \le t\}$. In this notation $K' = K_{\le \rho /2}$. Recall that Grünbaum’s Lemma (Lemma 4) guarantees that $\frac{1}{e} \le \frac{\text {Vol}_{n}(K_{\le 0})}{\text {Vol}_n(K)} \le 1-\frac{1}{e}$. Moreover, it is well known that the function $t \mapsto \text {Vol}_{n}(K_{\le t})^{1/n}$ is concave on its support, see again [27]. Then

$$\begin{aligned} \text {Vol}_n(K_{\le 0})^{1/n}\ge & {} \Big (\frac{1}{1+\rho /2}\Big ) \cdot \text {Vol}_n(K_{\le \rho /2})^{1/n} + \Big (\frac{\rho /2}{1+\rho /2}\Big ) \cdot \underbrace{\text {Vol}_n(K_{\le -1})^{1/n}}_{\ge 0} \\ {}\ge & {} \Big (\frac{1}{1+\rho /2}\Big ) \cdot \text {Vol}_n(K_{\le \rho /2})^{1/n} \end{aligned}$$

and so

$$\begin{aligned} \Big (1-\frac{1}{e}\Big ) \cdot \text {Vol}_n(K) \ge \text {Vol}_n(K_{\le 0}) \ge \Big (\frac{1}{1+\rho /2}\Big )^n \cdot \text {Vol}_n(K_{\le \rho /2}) \end{aligned}$$

Rearranging gives the first claim in the form $\text {Vol}_n(K_{\le \rho /2}) \le (1-\frac{1}{e}) \cdot (1+\frac{\rho }{2})^n \cdot \text {Vol}_n(K)$. For the 2nd part we verify that for $\rho \le \frac{1}{4n}$ one has $(1-\frac{1}{e}) \cdot (1+\frac{\rho }{2})^n \le (1-\frac{1}{e}) \cdot \exp (\frac{\rho }{2}) \le \frac{3}{4}$. $\square$

#### Lemma 13

Consider a call of $\textsc {Cut-Or-Average}$ on $(K,\Lambda )$ where $K \subseteq r B_2^n$ for some $r>0$. Then the total number of iterations of the outer WHILE loop over all recursion levels is bounded by $O(n^2 \log (\frac{nr}{\lambda _1(\Lambda )}))$.

#### Proof

**Fig. 3:**
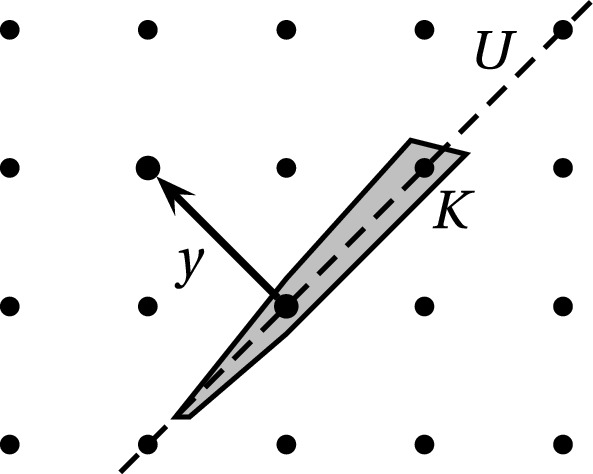
Visualization of lines (12)+(13) (with $n=2$ and $\Lambda =\mathbb {Z}^2 = \Lambda ^*$)

Consider any recursive run of the algorithm. The convex set will be of the form ${\tilde{K}}:= K \cap U$ and the lattice will be of the form ${\tilde{\Lambda }}:= \Lambda \cap U$ where *U* is a subspace and we denote ${\tilde{n}}:= \dim (U)$. We think of ${\tilde{K}}$ and ${\tilde{\Lambda }}$ as ${\tilde{n}}$-dimensional objects. Let ${\tilde{K}}_t \subseteq {\tilde{K}}$ be the convex body after *t* iterations of the outer WHILE loop. Recall that $\text {Vol}_{{\tilde{n}}}({\tilde{K}}_t) \le (\frac{3}{4})^t \cdot \text {Vol}_{{\tilde{n}}}({\tilde{K}})$ by Lemma 12 and $\text {Vol}_{{\tilde{n}}}({\tilde{K}}) \le r^{{\tilde{n}}} \text {Vol}_{{\tilde{n}}}(B_2^{{\tilde{n}}})$. Our goal is to show that for *t* large enough, there is a non-zero lattice vector $y \in {\tilde{\Lambda }}^*$ with $\Vert y\Vert _{({\tilde{K}}_t-{\tilde{K}}_t)^{\circ }} \le \frac{1}{2}$ which then causes the algorithm to recurse, see Fig. 3. To prove existence of such a vector *y*, we use Minkowski’s Theorem (Theorem 2) followed by the Blaschke-Santaló-Bourgain-Milman Theorem (Theorem 7) to obtain

$$\begin{aligned}{} & {} \lambda _1( {\tilde{\Lambda }}^*, ({\tilde{K}}_t-{\tilde{K}}_t)^{\circ }) {\mathop {\le }\limits ^{\text {Thm}~2}}2 \cdot \Big (\frac{\det ({\tilde{\Lambda }}^*)}{\text {Vol}_{{\tilde{n}}}( ({\tilde{K}}_t-{\tilde{K}}_t)^{\circ })}\Big )^{1/{\tilde{n}}} \\{} & {} \quad {\mathop {\le }\limits ^{\text {Thm}~7}} 2C \cdot \Big (\frac{\text {Vol}_{{\tilde{n}}}({\tilde{K}}_t-{\tilde{K}}_t)}{\det ({\tilde{\Lambda }}) \cdot \text {Vol}_{{\tilde{n}}}(B_2^{{\tilde{n}}})^2} \Big )^{1/{\tilde{n}}}\\{} & {} \quad {\mathop {\le }\limits ^{\text {Thm}~6}} 2 \cdot 4 \cdot \frac{\sqrt{{\tilde{n}}}}{2} \cdot C \Big (\frac{\text {Vol}_{{\tilde{n}}}({\tilde{K}}_t)}{\det ({\tilde{\Lambda }}) \cdot \text {Vol}_{{\tilde{n}}}(B_2^{{\tilde{n}}})} \Big )^{1/{\tilde{n}}}\\\le & {} 4C\sqrt{{\tilde{n}}} \cdot r \cdot \frac{(3/4)^{t/{\tilde{n}}}}{\det ({\tilde{\Lambda }})^{1/{\tilde{n}}}} \\\le & {} 4 C \cdot \frac{{\tilde{n}} \cdot r}{\lambda _1(\Lambda )} \cdot (3/4)^{t/{\tilde{n}}} \end{aligned}$$

Here we use the convenient estimate of $\text {Vol}_{{\tilde{n}}}(B_2^{{\tilde{n}}}) \ge \text {Vol}_{{\tilde{n}}}(\frac{1}{\sqrt{{\tilde{n}}}}B_{\infty }^{{\tilde{n}}}) = (\frac{2}{\sqrt{{\tilde{n}}}})^{{\tilde{n}}}$. Moreover, we have used that by Lemma 3 one has $\det ({\tilde{\Lambda }}) \ge (\frac{\lambda _1(\Lambda )}{\sqrt{{\tilde{n}}}})^{{\tilde{n}}}$. Then $t = \Theta ({\tilde{n}} \log (\frac{{\tilde{n}}r}{\lambda _1(\Lambda )}))$ iterations suffice until $\lambda _1( {\tilde{\Lambda }}^*, ({\tilde{K}}_t-{\tilde{K}}_t)^{\circ }) \le \frac{1}{2}$ and the algorithm recurses. Hence the total number of iterations of the outer WHILE loop over all recursion levels can be bounded by $O(n^2 \log (\frac{nr}{\lambda _1(\Lambda )}))$. $\square$

The iteration bound of Lemma 13 can be improved by amortizing the volume reduction over the different recursion levels following the approach of Jiang [30]. We refrain from that to keep our approach simple.

### Running times of the subroutines

We have already seen that the number of iterations of the Cut-or-Average algorithm is polynomially bounded. Goal of this subsection is to prove that all used subroutines can be implemented in time that is single-exponential or less. First we prove that steps (2)+(3) take polynomial time.

#### Lemma 14

For any convex body $K \subseteq \mathbb {R}^n$ one can compute the barycenter *c* and a $\varvec{0}$-centered ellipsoid  in randomized polynomial time so that .

#### Proof

We say that a convex body $Q \subseteq \mathbb {R}^n$ is *centered and isotropic* if the uniform random sample $X \sim Q$ satisfies the following conditions: (i) $\mathop {\mathbb {E}}[X]=\varvec{0}$ and (ii) $\mathop {\mathbb {E}}[XX^T] = I_n$. For any convex body *K* one can compute an affine linear transformation $T: \mathbb {R}^n \rightarrow \mathbb {R}^n$ in polynomial time^1^ so that *T*(*K*) is centered and isotropic; this can be done for example by obtaining polynomially many samples of *X*, see [31, 32]. A result by Kannan, Lovász and Simonovits (Lemma 5.1 in [31]) then says that any such centered and isotropic body *T*(*K*) satisfies $B_2^n \subseteq T(K) \subseteq (n+1) B_2^n$. Then $c:= T^{-1}(\varvec{0})$ and  satisfy the claim. $\square$

**Fig. 4:**
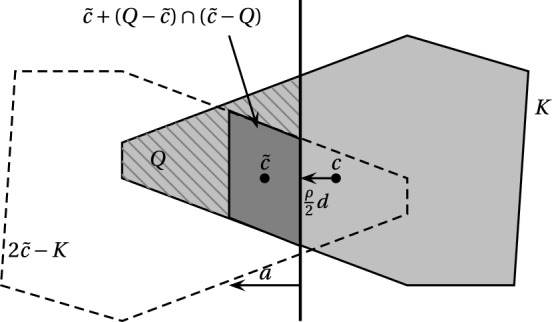
Visualization of the proof of Lemma 15 where ${\tilde{c}} = c + \rho d$

In order for the call of $\textsc {ApxIP}$ in step (7) to be efficient, we need that the symmetrizer of the set is large enough volume-wise, see Theorem 1. We will prove now that this is indeed the case. In particular for any parameters $2^{-\Theta (n)} \le \rho \le 0.99$ and $R \le 2^{O(n)}$ we will have $\text {Vol}_n((Q-{\tilde{c}}) \cap ({\tilde{c}}-Q)) \ge 2^{-\Theta (n)} \text {Vol}_n(Q)$ which suffices for our purpose (Fig. 4).

#### Lemma 15

In step (7), the set $Q:= \{ x \in K \mid \left<a,x\right> \ge \left<a,c+\frac{\rho }{2} d\right>\}$ and the point ${\tilde{c}}:= c+\rho d$ satisfy $\text {Vol}_n((Q-{\tilde{c}}) \cap ({\tilde{c}}-Q)) \ge (1-\rho )^n \cdot \frac{\rho }{2R} \cdot 2^{-n} \cdot \text {Vol}_n(Q)$.

#### Proof

Consider the symmetrizer $K':= (K-c) \cap (c-K)$ which has $\text {Vol}_n(K') \ge 2^{-n}\text {Vol}_n(K)$ by Theorem 5 as *c* is the barycenter of *K*. Set $K'':= \{ x \in K' \mid |\left<a,x\right>| \le \frac{\rho }{2}|\left<a,d\right>|\}$. As $K'$ is symmetric and all $x \in K'$ satisfy $|\left<a,x\right>| \le R|\left<a,d\right>|$, we have $\text {Vol}_n(K'') \ge \frac{\rho }{2R} \text {Vol}_n(K')$. Now consider

$$\begin{aligned} P:= & {} (1-\rho )(K''+c) + \rho (c+d) \\= & {} (1-\rho ) K'' + (c+\rho d) \\{} & {} {\mathop {\subseteq }\limits ^{(*)}} K \cap \big \{ x \in \mathbb {R}^n: \left<a,x\right> \ge \big <a,c + \frac{\rho }{2}d\big >\big \} = Q. \end{aligned}$$

For the inclusion in $(*)$ we use that $K'' + c \subseteq K$ and $c+d \in K$; moreover for any $x \in K''$ one has $\left<a,(1-\rho )x+c+\rho d\right> \ge \left<a,c+\frac{\rho }{2}d\right>$. Finally, *P* is symmetric about $c+\rho d$ and hence

$$\begin{aligned} \text {Vol}_n((Q-{\tilde{c}}) \cap ({\tilde{c}}-Q)) \ge \text {Vol}_n(P) \ge (1-\rho )^n \cdot \frac{\rho }{2R} \cdot 2^{-n} \cdot \text {Vol}_n(Q) \end{aligned}$$

as $$Q \subseteq K$$. $$\square $$

Step (10) can be done in polynomial time and we defer the analysis to Sect. 4. Step (12) corresponds to finding a shortest non-zero vector in a lattice w.r.t. norm $\Vert \cdot \Vert _{(K-K)^{\circ }}$ which can be done in time $2^{O(n)}$ using the Sieving algorithm [33].

### Conclusion on the cut-or-average algorithm

From the discussion above, we can summarize the performance of the algorithm in Fig. 1 as follows:

#### Theorem 16

Given a full rank matrix $B \in \mathbb {Q}^{n \times n}$ and parameters $r>0$ and $\ell \ge 5(n+1)$ with $\ell \in \mathbb {N}$ and a separation oracle for a closed convex set $K \subseteq r B_2^n$, there is a randomized algorithm that with high probability finds a point $x \in K \cap \frac{1}{\ell }\Lambda (B)$ or decides that $K \cap \Lambda (B) = \emptyset$. Here the running time is $2^{O(n)}$ times a polynomial in $\log (r)$ and the encoding length of *B*.

This can be easily turned into an algorithm to solve integer linear programming:

#### Theorem 17

Given a full rank matrix $B \in \mathbb {Q}^{n \times n}$, a parameter $r>0$ and a separation oracle for a closed convex set $K \subseteq r B_2^n$, there is a randomized algorithm that with high probability finds a point $x \in K \cap \Lambda (B)$ or decides that there is none. The running time is $2^{O(n)}n^n$ times a polynomial in $\log (r)$ and the encoding length of *B*.

#### Proof

Suppose that $K \cap \Lambda \ne \emptyset$ and fix an (unknown) solution $x^* \in K \cap \Lambda$. We set $\ell := \lceil 5(n+1) \rceil$. We iterate through all $v \in \{ 0,\ldots ,\ell -1\}^n$ and run Theorem 16 on the set *K* and the shifted lattice $v+\ell \Lambda$. For the outcome of *v* with $x^* \equiv v \mod \ell$ one has $K \cap (v+\ell \Lambda ) \ne \emptyset$ and so the algorithm will discover a point $x \in K \cap (v+\Lambda )$. $\quad \square$

## An asymmetric approximate Carathéodory theorem

In this section we show correctness of (10) and prove that given lattice points $X \subseteq \Lambda$ that are contained in a in a 3-scaling of *K* and satisfy $c \in \text {conv}(X)$, we can find a point in $\frac{\Lambda }{\ell } \cap K$. The *Approximate Carathéodory Theorem* states the following.

Given any point-set $X \subseteq B_2^n$ in the unit ball with $\varvec{0} \in \text {conv}(X)$ and a parameter $k \in \mathbb {N}$, there exist $u_1,\ldots ,u_k \in X$ (possibly with repetition) such that

$$\begin{aligned} \left\| \frac{1}{k}\sum _{i=1}^k u_i\right\| _2 \le O\left( 1/\sqrt{k}\right) . \end{aligned}$$

The theorem is proved, for example, by Novikoff [8] in the context of the *perceptron algorithm*. An $\ell _p$-version was provided by Barman [9] to find Nash equilibria. Deterministic and nearly-linear time methods to find the convex combination were recently described in [10]. In the following, we provide a generalization to asymmetric convex bodies and the dependence on *k* will be weaker but sufficient for our analysis of our Cut-or-Average algorithm from Sect. 3.

Recall that with a symmetric convex body *K*, we one can associate the *Minkowski norm* ${\Vert \cdot \Vert _K}$ with $\Vert x\Vert _K = \inf \{ s \ge 0 \mid x \in sK\}$. In the following we will use the same definition also for an arbitrary convex set *K* with $\varvec{0} \in K$. Symmetry is not given but one still has $\Vert x+y\Vert _K \le \Vert x\Vert _K + \Vert y\Vert _K$ for all $x,y \in \mathbb {R}^n$ and $\Vert \alpha x\Vert _K = \alpha \Vert x\Vert _K$ for $\alpha \in \mathbb {R}_{\ge 0}$. Using this notation we can prove the main result of this section.

### Lemma 18

Given a point-set $X \subseteq K$ contained in a convex set $K \subseteq {\mathbb {R}}^n$ with $\varvec{0} \in \text {conv}(X)$ and a parameter $k \in \mathbb {N}$, there exist $u_1,\ldots ,u_k \in X$ (possibly with repetition) so that

$$\begin{aligned} \left\| \frac{1}{k}\sum _{i=1}^k u_i\right\| _K \le {\min \{|X|,n+1\}}/ {k}. \end{aligned}$$

Moreover, given *X* as input, the points $u_1,\ldots ,u_k$ can be found in time polynomial in |*X*|, *k* and *n*.

### Proof

Let $\ell = \min \{|X|, n+1\}$. The claim is true whenever $k \le \ell$ since then we may simply pick an arbitrary point in *X*. Hence from now on we assume $k>\ell$.

By Carathéodory’s theorem, there exists a convex combination of zero, using $\ell$ elements of *X*. We write $\varvec{0} = \sum _{i=1}^\ell \lambda _i v_i$ where $v_i \in X$, $\lambda _i \ge 0$ for $i \in [\ell ]$ and $\sum _{i=1}^\ell \lambda _i = 1$. Consider the numbers $L_i = (k-\ell ) \lambda _i +1$. Clearly, $\sum _{i=1}^\ell L_i = k.$ This implies that there exists an integer vector $\mu \in {\mathbb {N}}^\ell$ with $\mu \ge (k-\ell ) \lambda$ and $\sum _{i=1}^\ell \mu _i = k$. It remains to show that we have

$$\begin{aligned} \left\| \frac{1}{k}\sum _{i=1}^\ell \mu _i v_i \right\| _K \le \ell / {k}. \end{aligned}$$

In fact, one has

$$\begin{aligned} \Big \Vert \sum _{i=1}^\ell \mu _iv_i\Big \Vert _K= & {} \Big \Vert \sum _{i=1}^\ell \underbrace{(\mu _i-(k-\ell )\lambda _i)}_{\ge 0}v_i +\underbrace{(k-\ell )}_{\ge 0} \sum _{i=1}^\ell \lambda _iv_i\Big \Vert _K \\\le & {} \sum _{i=1}^\ell (\mu _i-(k-\ell )\lambda _i) \underbrace{\Vert v_i\Vert _K}_{\le 1} + (k-\ell ) \underbrace{\Big \Vert \sum _{i=1}^\ell \lambda _iv_i\Big \Vert _K}_{=0}\\\le & {} \ell . \end{aligned}$$

For the moreover part, note that the coefficients $\lambda _1,\ldots ,\lambda _{\ell }$ are the extreme points of a linear program which can be found in polynomial time. Finally, the linear system $\mu \ge \lceil (k-\ell ) \lambda \rceil , \sum _{i=1}^{\ell } \mu _i = k$ has a totally unimodular constraint matrix and the right hand side is integral, hence any extreme point solution is integral as well, see e.g. [29]. $\square$

### Lemma 19

For any integer $\ell \ge 5(n+1)$, the convex combination $\mu$ computed in line (10) satisfies $\sum _{x \in X} \mu _x x \in K$.

### Proof

We may translate the sets *X* and *K* so that $c=\varvec{0}$ without affecting the claim. Recall that $z \in \text {conv}(X)$. By Carathéodory’s Theorem there are $v_1,\ldots ,v_m \in X$ with $m \le n+1$ so that $z \in \text {conv}\{v_1,\ldots ,v_m\}$ and so $\varvec{0} \in \text {conv}\{ v_1-z,\ldots ,v_m-z\}$. We have $v_i \in 3K$ by Lemma 9 and  as well as $z \in \frac{1}{4}K$. Hence $\Vert v_i-z\Vert _K \le \Vert v_i\Vert _K + \Vert -z\Vert _K \le \frac{13}{4}$. We apply Lemma 18 and obtain a convex combination $\mu \in \frac{\mathbb {Z}_{\ge 0}^m}{\ell }$ with $\Vert \sum _{i=1}^m \mu _i(v_i-z)\Vert _{\frac{13}{4}K} \le \frac{m}{\ell }$. Then

$$\begin{aligned} \Big \Vert \sum _{i=1}^m \mu _i v_i\Big \Vert _K \le \Big \Vert \sum _{i=1}^m \mu _i(v_i-z)\Big \Vert _K + \underbrace{\Vert z\Vert _K}_{\le 1/4} \le \frac{13}{4} \frac{m}{\ell } + \frac{1}{4} \le 1 \end{aligned}$$

if $\ell \ge \frac{13}{3}m$. This is satisfies if $\ell \ge 5(n+1)$. $\square$

## IPs with polynomial variable range

Now we come to our second method that reduces (IP) to (Approx-IP) that applies to integer programming in *standard equation form*

2 $$\begin{aligned} Ax = b, \,x \in \mathbb {Z}^n, \, 0 \le x_i\le u_i, \, i=1,\dots ,n, \end{aligned}$$

Here, $A \in {\mathbb {Z}}^{m \times n}$, $b \in {\mathbb {Z}}^m$, and the $u_i\in {\mathbb {N}}_{+}$ are positive integers that bound the variables from above. Our main goal is to prove the following theorem.

### Theorem 20

The integer feasibility problem (2) can be solved in time $2^{O(n)} \prod _{i=1}^n \log _2 (u_i+1)$.

We now describe the algorithm. It is again based on the approximate integer programming technique of Dadush [7]. We exploit it to solve integer programming exactly via the technique of *reflection sets* developed by Cook et al. [34]. For each $i = 1,\dots ,n$ we consider the two families of hyperplanes that slice the feasible region with the shifted lower and upper bounds respectively

3 $$\begin{aligned} x_i = 2^{j-1} \text { and } x_i = u_i - 2^{j-1}, \, 0 \le j \le \lceil \log _2(u_i)\rceil . \end{aligned}$$

**Fig. 5:**
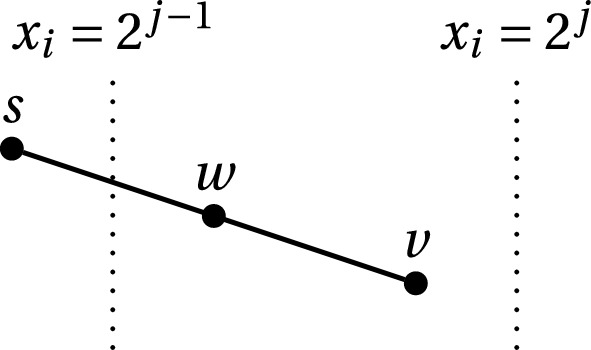
The reflection set

Following [34], we consider two points *w*, *v* that lie in the region between two consecutive planes $x_i = 2^{j-1}$ and $x_i = 2^{j}$ for some *j*, see Fig. 5.

Suppose that $w_i \le v_i$ holds. Let *s* be the point such that $w = 1/2(s + v)$. The line-segment *s*, *v* is the line segment *w*, *v* scaled by a factor of 2 from *v*. Let us consider what can be said about the *i*-th component of *s*. Clearly $s_i \ge 2^{j-1} - (2^j -2^{j-1}) = 0$. Similarly, if *w* and *v* lie in the region in-between $x_i = 0$ and $x_i = 1/2$, then $s_i \ge -1/2$. We conclude with the following observation.

### Lemma 21

Consider the hyperplane arrangement defined by the equations (3) as well as by $x_i = 0$ and $x_i = u_{i}$ for $1\le i\le n$. Let $K\subseteq {\mathbb {R}}^n$ a cell of this hyperplane arrangement and $v \in K$. If $K'$ is the result of scaling *K* by a factor of 2 from *v*, i.e.

$$\begin{aligned} K' = \{ v + 2(w-v) \mid w \in K\}, \end{aligned}$$

then $K'$ satisfies the inequalities $-1/2\le x_i \le u_i + 1/2$ for all $1\le i\le n$.

We use this observation to prove Theorem 20:

### Proof of Theorem 20

**Fig. 6:**
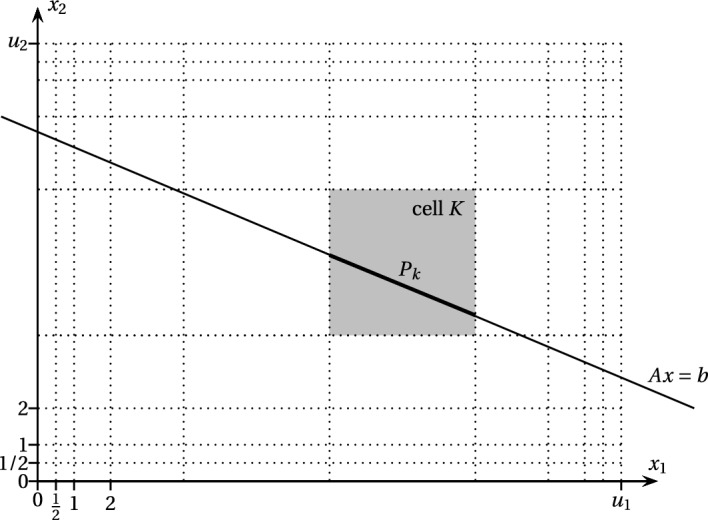
Visualization of the proof of Theorem 20

The task of (2) is to find an integer point in the affine subspace defined by the system of equations $Ax = b$ that satisfies the bound constraints $0\le x_i \le u_i$. We first partition the feasible region with the hyperplanes (3) as well as $x_i = 0$ and $x_i = u_i$ for each *i*. We then apply the approximate integer programming algorithm with approximation factor 2 on each convex set $P_K = \{x \in \mathbb {R}^n \mid Ax = b\}\cap K$ where *K* ranges over all cells of the arrangement (see Fig. 6). In $2^{O(n)}$ time, the algorithm either finds an integer point in the convex set $C_K$ that results from $P_K$ by scaling it with a factor of 2 from its center of gravity, or it asserts that $P_K$ does not contain an integer point. Clearly, $C_K \subseteq \{x \in \mathbb {R}^n \mid Ax = b\}$ and if the algorithm returns an integer point $x^*$, then, by Lemma 21, this integer point also satisfies the bounds $0\le x_i \le u_i$. The running time of the algorithm is equal to the number of cells times $2^{O(n)}$ which is $2^{O(n)} \prod _{i=1}^n \log _2 (u_i+1)$. $\square$

### IPs in inequality form

We can also use Theorem 20 to solve integer linear programs in *inequality form*. Here the efficiency is strongly dependent on the number of inequalities.

#### Theorem 22

Let $A \in \mathbb {Q}^{m \times n}$, $b \in \mathbb {Q}^{m}$, $c \in \mathbb {Q}^n$ and $u \in \mathbb {N}_+^n$. Then the integer linear program

$$\begin{aligned} \max \big \{ \left<c,x\right> \mid Ax \le b, \; 0 \le x \le u, \; x \in \mathbb {Z}^n \big \} \end{aligned}$$

can be solved in time $n^{O(m)} \cdot (2\log (1+\Delta ))^{O(n+m)}$ where $\Delta := \max \{ u_i \mid i=1,\ldots ,n\}$.

#### Proof

Via binary search it suffices to solve the feasibility problem

4 $$\begin{aligned} \left<c,x\right> \ge \gamma , \; Ax \le b, \; 0 \le x \le u, \; x \in \mathbb {Z}^n \end{aligned}$$

in the same claimed running time. We apply the result of Frank and Tardos (Theorem 8) and replace $c,\gamma ,A,b$ by integer-valued objects of bounded $\Vert \cdot \Vert _{\infty }$-norm so that the feasible region of (4) remains the same. Hence we may indeed assume that $c \in \mathbb {Z}^n$, $\gamma \in \mathbb {Z}$, $A \in \mathbb {Z}^{m \times n}$ and $b \in \mathbb {Z}^m$ with $\Vert c\Vert _{\infty },|\gamma |,\Vert A\Vert _{\infty }, \Vert b\Vert _{\infty } \le 2^{O(n^3)} \cdot \Delta ^{O(n^2)}$. Any feasible solution *x* to (4) has a slack bounded by $\gamma -\left<c,x\right> \le |\gamma | + \Vert c\Vert _{\infty } \cdot n \cdot \Delta \le N$ where we may choose $N:= 2^{O(n^3)} \Delta ^{O(n^2)}$. Similarly $b_i-\left<A_i,x\right> \le N$ for all $i \in [n]$. We can then introduce slack variables $y \in \mathbb {Z}_{\ge 0}$ and $z \in \mathbb {Z}_{\ge 0}^m$ and consider the system

5 $$\begin{aligned} \begin{array}{lll} \left<c,x\right> + y = \gamma , &{} Ax + z = b, &{} \\ 0 \le x \le u, &{} 0\le y \le N, &{} 0 \le z_j \le N \; \forall j \in [m], \\ (x,y,z) \in \mathbb {Z}^{n+1+m} &{} &{} \end{array} \end{aligned}$$

in equality form which is feasible if and only if (4) is feasible.

Then Theorem 20 shows that such an integer linear program can be solved in time

$$\begin{aligned} 2^{O(n+m)} \cdot \Big (\prod _{i=1}^n \ln (1+u_i) \Big ) \cdot (\ln (1+N))^{m+1} \le n^{O(m)} \cdot (2\log (1+\Delta ))^{O(n+m)}. \end{aligned}$$

$$\square $$

### Subset sum and knapsack

The *subset-sum problem (with multiplicities)* is an integer program of the form (2) with one linear constraint. Polak and Rohwedder [35] have shown that subset-sum with multiplicities — that means $\sum _{i=1}^n x_iz_i=t, 0 \le x_i \le u_i \; \forall i \in [n], x \in \mathbb {Z}^n$ — can be solved in time $O(n +z_{\max }^{5/3})$ times a polylogarithmic factor where $z_{\max }:= \max _{i=1,\ldots ,n} z_i$. The algorithm of Frank and Tardos [28] (Theorem 8) finds an equivalent instance in which $z_{\max }$ is bounded by $2^{O(n^3)} u_{\max }^{O(n^2)}$. All-together, if each multiplicity is bounded by a polynomial *p*(*n*), then the state-of-the-art for subset-sum with multiplicities is straightforward enumeration resulting in a running time $n^{O(n)}$ which is the current best running time for integer programming. We can significantly improve the running time in this regime. This is a direct consequence of Theorem 22.

#### Corollary 23

The subset sum problem with multiplicities of the form $\sum _{i=1}^n x_iz_i=t, 0 \le x \le u, x \in \mathbb {Z}^n$ can be solved in time $2^{O(n)} \cdot (\log (1+\Vert u\Vert _{\infty }))^n$. In particular if each multiplicity is bounded by a polynomial *p*(*n*), then it can be solved in time $(\log n )^{O(n)}$.

*Knapsack* with multiplicities is the following integer programming problem

6 $$\begin{aligned} \max \big \{\left<c, x\right> \mid x \in {\mathbb {Z}}_{\ge 0}^n, \, \left<a,x\right> \le \beta , 0 \le x\le u \big \}, \end{aligned}$$

where $c, a,u \in {\mathbb {Z}}_{\ge 0}^n$ are integer vectors. Again, via the preprocessing algorithm of Frank and Tardos [28] (Theorem 8) one can assume that $\Vert c\Vert _\infty$ as well as $\Vert a\Vert _\infty$ are bounded by $2^{O(n^3)} u_{\max }^{O(n^2)}$. If each $u_i$ is bounded by a polynomial in the dimension, then the state-of-the-art^2^ for this problem is again straightforward enumeration which leads to a running time of $n^{O(n)}$. Also in this regime, we can significantly improve the running time which is an immediate consequence of Theorem 22.

#### Corollary 24

A knapsack problem (6) can be solved in time $2^{O(n)} \cdot (\log (1+\Vert u\Vert _{\infty }))^n$. In particular if $\Vert u\Vert _\infty$ is bounded by a polynomial *p*(*n*) in the dimension, it can be solved in time $(\log n)^{O(n)}$.

## Footnotes

1 At least up to negligible error terms.

2 At least at the time of the first submission of this paper.
